# Speech and music source separation for cochlear implant users: front-end and end-to-end approach

**DOI:** 10.3389/fnins.2025.1696669

**Published:** 2026-01-13

**Authors:** Sina Tahmasebi, Waldo Nogueira

**Affiliations:** 1 Department of Otolaryngology, Hannover Medical School, Hanover, Germany; 2 Cluster of Excellence “Hearing4all”, Hannover, Germany; 3 Department of Microelectronics and Electronic Systems, Institute of Neuroscience, Universitat Automoma de Barcelona, Barcelona, Spain

**Keywords:** cochlear implants, DNNs, end-to-end source separation, singing music, speech

## Abstract

A cochlear implant (CI) is a surgically implanted neuroprosthetic device designed to restore auditory perception in individuals with profound sensorineural hearing loss. While CI users generally demonstrate good speech intelligibility in quiet listening environments, their performance significantly declines in the presence of competing sound sources. Moreover, music perception and appreciation remain limited for many CI users. These limitations are largely attributed to the inadequate representation of pitch information, which is critical for both music and speech stream segregation in complex auditory scenes. To address these challenges, source separation techniques have been increasingly employed to enhance target speech and isolate singing voices in music. Previous research has shown that CI users report greater music enjoyment when vocals are enhanced relative to the accompanying background instrumentation. Building on this, recent studies have leveraged deep neural networks (DNNs) as both front-end and end-to-end modules to improve speech intelligibility and music enjoyment for CI users. In the present study, we compare front-end and end-to-end DNN-based source separation approaches for two tasks: speech masked by competing speech and singing music. All implemented pipelines were first evaluated using objective instrumental metrics. Based on these results, the models were subsequently assessed in a listening experiment involving nine bilateral CI users. While the end-to-end pipeline outperformed the front-end pipeline in speech understanding tasks, the front-end approach yielded higher scores in music appreciation questionnaires. These findings support the hypothesis that CI sound coding strategies can be effectively combined with DNN-based source separation models. Furthermore, we hypothesize that the limited performance of end-to-end music source separation in enhancing music perception for CI users may be due to the absence of a dedicated sound coding strategy tailored for instrumental music.

## 1 Introduction

Cochlear implantation is a widely accepted and clinically effective intervention for the rehabilitation of individuals with severe to profound sensorineural hearing loss. Cochlear implant (CI) recipients usually exhibit high levels of speech understanding in quiet listening conditions within a few days to weeks following the implantation. CI sound coding strategies are responsible for converting acoustic signals into electrical pulses that are used to stimulate the cochlea and evoke the perception of sound. Four of the most widely adopted sound coding strategies are the Advanced Combination Encoder (ACE) (Holden et al., 2002), Continuous Interleaved Sampling (Wilson et al., 1993), the High Resolution Strategy (Koch et al., 2007; Nogueira et al., 2009, and Fine Structure Processing (Hochmair et al., 2015; Arnoldner et al., 2007) which have been in use for more than a decade. These sound coding strategies rely on traditional signal processing algorithms to generate electrical pulses that are intelligible to CI users. The resulting patterns of electrical stimulation, when visualized, are referred to as electrodograms. Over the years, various enhancements have been introduced to individual components of the signal processing chain. However, there remains a strong demand for further improvements to enhance speech understanding in the presence of competing sound sources (Wouters et al., 2015) or competing talker (El Boghdady et al., 2019) and music appreciation (Nogueira et al., 2018).

This demand has driven a significant transformation in CI sound coding strategies, increasingly incorporating deep learning techniques and shifting toward deep learning-based solutions rather than classic signal processing approaches. This growing emphasis on deep learning techniques has become a prominent focus of recent research. Deep neural network (DNN) models used to improve the CI experience are typically applied either in a front-end configuration, where the model is positioned at the very beginning of the processing pipeline, or in a end-to-end configuration, where the model is integrated within the sound coding strategy at one of the later stages. While front-end source separation pipelines focus on isolating individual sources, end-to-end approaches for CI users are designed to directly predict a single clean target signal. Although DNN models have the potential to be incorporated directly into the sound coding strategy, they have less often been utilized in this manner. Goehring et al. (2017) integrated a DNN to estimate a gain function, aiming at enhanced envelopes in the CI sound coding strategy and observed significant improvements speech reception threshold in noisy conditions. For hearing aids, a real-time, single-microphone deep learning front-end algorithm that restores speech intelligibility in noisy environments to near normal-hearing levels optimized via novel intelligibility metrics has been proposed and commercialized (Diehl et al., 2023, 2022). Therefore there is great potential to for DNN Technology to improve speech understanding and music perception through DNN Technology. DNN-based front-end approaches have been widely used for CI users, both for target enhancement and source separation, to improve speech understanding (Lai et al., 2016) as well as music perception and appreciation (Tahmasebi et al., 2020; Gajęcki and Nogueira, 2018). However, end-to-end and back-end approaches have been used only for speech denoising tasks (Zheng et al., 2021; Gajecki et al., 2023; Huang et al., 2023) and have not been investigated for source separation tasks, including music source separation, for CI users.

Recent studies in the field of audio signal processing have demonstrated the strong performance of time-domain deep learning approaches, such as TasNet (Luo and Mesgarani, 2018), in the context of speech enhancement and source separation. These algorithms aim to enhance the intelligibility of a target speech signal in the presence of competing sound sources. Within this line of research, which are based on temporal convolutional networks (TCNs) (Lea et al., 2016), time-domain models like Conv-TasNet have been shown to outperform or at least obtain similar performance to traditional frequency-domain methods, offering superior results in isolating and enhancing target speech (Luo and Mesgarani, 2018). In contrast to conventional Short-Time Fourier Transform (STFT) domain approaches, time-domain approaches are capable of capturing both magnitude and phase information, enabling direct optimization with respect to loss functions in both the time and frequency domains (Fu et al., 2018; Gusó et al., 2022).

One of the primary focuses of DNN applications for CI users has been speech denoising or de-reverberation to improve speech comprehension in noisy environments. These models typically generate a single output aimed at predicting a denoised target signal, thereby enhancing speech intelligibility. While speech enhancement methods focus on extracting high-quality, intelligible speech from noisy inputs, audio source separation models are designed to disentangle multiple sound sources for further manipulation. These models can apply targeted enhancement to one or more sources while preserving the others. In the context of music, source separation allows selective emphasis of individual components within an audio mixture, such as enhancing vocals or emphasizing specific spectral regions like bass or mid frequencies and thereby improving music appreciation for CI users (Althoff et al., 2024). In the context of music, studies have shown that CI users experience greater enjoyment when vocals are enhanced relative to background instruments (Buyens et al., 2014; Pons et al., 2016). Achieving this enhancement requires separating the vocal track from the background instruments, a task well suited to source separation models. Moreover, reducing spectral complexity using front-end algorithm (Nagathil et al., 2015) or reducing the number of selected channel for stimulation (Tahmasebi et al., 2023) has been shown to have a positive impact on music appreciation among CI users, an effect that can be also achieved through source separation methods (Gauer et al., 2022). Similarly, in speech-related applications, considering that understanding speech masked by other speech seem to be more challenging for CI users (Başkent and Gaudrain, 2016), there are situations in which two or more voices must be preserved or selectively enhanced to meet user needs.

Recent studies have also demonstrated the advantages of back-end (Huang et al., 2023) and end-to-end (Gajecki et al., 2023) approaches over front-end algorithms for speech denoising tasks in CI users. The present study aims to investigate the efficacy of front-end and end-to-end pipelines for speech and music source separation tasks. The end-to-end pipeline offers advantages such as reduced algorithmic latency and the avoidance of redundant computational steps. In contrast, the front-end pipeline provides full access to and control over the raw audio input, which is advantageous for tasks requiring direct signal manipulation.

For music enhancement, and building on previous research showing that front-end enhancement of vocals relative to background instruments can significantly improve music appreciation in CI users (Buyens et al., 2014; Tahmasebi et al., 2020), we introduce the parameter VIR_eqi_. This parameter denotes the level of vocal-to-instrument ratio enhancement applied within the end-to-end processing framework. Importantly, VIR_eqi_ is defined such that it is directly comparable to the VIR manipulation implemented at the front-end, thereby ensuring an equivalent enhancement magnitude across both processing pipelines. To evaluate the effectiveness of both approaches, we conducted listening experiments with CI users, assessing the performance of the front-end and end-to-end pipelines in speech understanding tasks as well as in terms of lyric intelligibility and overall impression of singing music pieces.

## 2 Methods and materials

### 2.1 Participants

Nine bilateral CI users, each implanted with a Nucleus device with at least a year of CI experience, participated in the perceptual experiments. Table 1 presents the demographic characteristics of the participants. All participants provided written informed consent in accordance with the guidelines of the Ethics Committee of the Hannover Medical School. Unilateral CI users with a functioning contralateral ear, CI users younger than 18 years, and individuals with less than one year of CI experience were excluded.

**Table 1:** Details of the CI subjects who participated in the experiments.

**Subject ID**	**Age (Y)**	**Gender**	**Cause of deafness**	**Duration of deafness (Y)**	**Tested side**	**CI experience on tested side (Y)**	**Tested iput SIR (dB)**
S01	66	F	Sudden deafness	8	R	15	5
S02	75	M	Unknown	6	L	9	0
S03	78	M	Sudden deafness	1	R	7	5
S04	73	M	Otosclerosis cochleae	1	R	21	5
S05	46	F	Unknown	4	R	1	5
S06	48	F	Unknown	0	R	1	5
S07	40	M	Otosclerosis cochleae	14	L	8	5
S08	54	F	Unknown	1	L	13	5
S09	73	F	Sudden deafness	0	R	9	5

For each subject, SIR denotes the input signal-to-interference ratio. Temporal data are reported in years (Y).

### 2.2 Advanced combination encoder sound coding strategy

**Figure 1:**
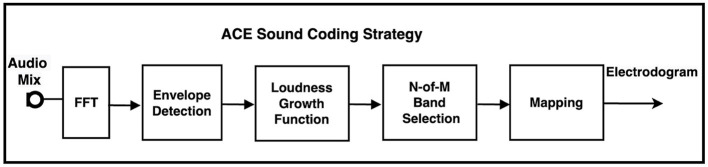
Block diagram of research advanced combination encoder (ACE) sound coding strategy.

The ACE (Cochlear Ltd, Sydney) sound coding strategy is one of the most established and widely implemented approaches in CI systems. These strategies are responsible for transforming the acoustic signals captured by the microphone into electrical stimulation patterns, or electrodograms, which are then delivered to the auditory nerve by the CI electrodes. ACE is one of CI sound coding strategies that employs, in its signal processing chain (Figure 1), a channel selection mechanism (N-of-M) based on spectral features of the input signal. For each frame of the audio signal, N (typically 8) channels out of M available channels (typically 22) are stimulated in a sequential manner, completing one full stimulation cycle per frame. The number of such cycles executed per second defines the channel stimulation rate. In the first step, the acoustic signal captured by the microphone is digitized at a sampling rate of 16 kHz and processed through an adaptive gain control (AGC) system to normalize and compress the input acoustic dynamic range. The signal is then passed through a filter bank, implemented via a Fast Fourier Transform (FFT), to analyze its spectral content. The envelope of each spectral band is estimated by computing the magnitude of the corresponding complex FFT coefficients. These spectral envelopes are subsequently subjected to a non-linear compression called loudness growth function (LGF). Finally, in a user-specific mapping step, the output of LGF block is mapped to the limited electrical dynamic range (EDR) available to each individual CI user. The EDR is defined as the subject's dynamic range between threshold level (THL) and most comfort level (MCL) in Cochlear's current units for electrical stimulation Nogueira et al. (2005).

### 2.3 Dataset

#### 2.3.1 Speech dataset

The separation of female-male sources was performed using front-end and end-to-end models trained on the LibriVox dataset (Beilharz et al., 2019) and tested on the Hochmair, Schulz, and Moser (HSM) dataset (Hochmair-Desoyer et al., 1997). The original LibriVox corpus consists of 110 h of audio material read by volunteers from German audiobooks. However, we used a subset of this corpus that included of 24 male and 24 female speakers, with each speaker contributing 70 sentences with a total duration of approximately 18 h. The quality of the audio material was assessed through manual evaluation, which indicated that the recordings were generally clear and consisted of read speech with minimal disfluencies. The HSM sentence test consists of 30 lists, each comprising 20 commonly used sentences, with a total of 106 words per list. The test includes recordings from both a female and a male speaker. All data sets were remixed at various signal-to-interference ratios (SIRs) for male and female voices.

#### 2.3.2 Singing music dataset

To train and test the front-end and end-to-end models for singing music source separation, MUSDB dataset (Rafii et al., 2017) was used. The MUSDB dataset is a publicly available collection comprising 150 professionally mixed songs (approximately 10 h in total duration) across various genres that is partitioned into a training set and a testing set, consisting of 100 and 50 songs, respectively. Each track includes four stereo source stems, bass, drums, vocals, and a composite of other instruments that combine to form a complete mix. The music tracks were segmented into 30-s excerpts, and the instrument stems were combined to create a two-track dataset consisting of isolated vocals and background instruments.

### 2.4 Front-end pipeline

**Figure 2:**
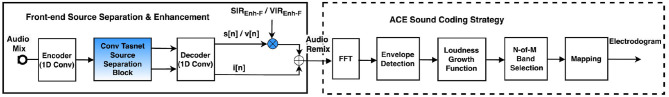
Block diagram of the cochlear implant sound coding Front-end pipeline. The source separation module has been integrated in the most upstream stage of the pipeline. SIR_Enh − F_ represents signal to interference ratio which aim to reduce or completely discard the interferer and VIR_Enh − F_ represents the front-end vocals-to-instruments enhancement applied to the predicted vocals. *s*[*n*], *v*[*n*], and *i*[*n*] denote the predicted speech, vocals and background interfere or background instruments, respectively, with *n* representing the sample index.

The front-end pipeline (Figure 2) in this study employs a DNN model introduced by Luo and Mesgarani (2018), known as Conv-TasNet. Similar to the model used in Luo and Mesgarani (2018), Conv-TasNet used in this study consist of three main signal processing blocks: the Encoder that encodes the audio into the intermediate domain (replacing the FFT in FFT based models), the separation block, that separates the sound sources, and the decoder block, that decodes the audio into the audio domain. Two front-end DNN models were independently trained to perform the two source separation tasks: (1) separation of female and male voices, and (2) separation of vocals from background instruments. Both models were optimized using the scale-invariant signal-to noise ratio (SI-SNR) (Le Roux et al., 2019) loss function, which minimizes the difference between the predicted audio outputs and their corresponding target signals. Table 2 presents the architectures of the DNN models used in this study. The Adam optimizer (Kingma and Ba, 2014) with an initial learning rate of 0.001 and max learning rate of 100 epochs were used to train the model.

**Table 2:** The hyperparameters of the networks utilized for training both the front-end and end-to-end models.

**Network hyperparameter**	**Speech source separation end-to-end**	**Speech source separation front-end**	**Music source separation end-to-end**	**Music source separation front-end**
N	128	128	128	128
L	16	16	16	16
B	64	64	128	128
H	128	128	256	256
S	64	128	64	128
P	3	3	3	3
X	8	8	8	8
R	3	3	3	3
Total number of parameters	∽ 833k	∽ 824k	∽ 2.470M	∽ 2.431M

### 2.5 End-to-end pipeline

**Figure 3:**
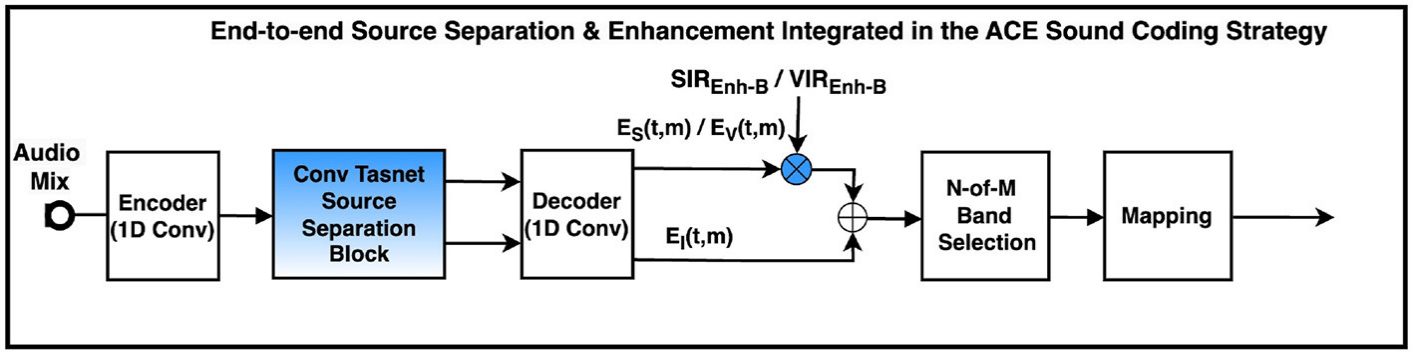
Block diagram of the cochlear sound coding end-to-end pipeline. The source separation module has replaced part of the ACE sound coding strategy and has been integrated at the most downstream stage of the processing pipeline. SIR_Enh − E_ and VIR_Enh − E_ represent the end-to-end signal to interferer ratio enhancement and vocals-to-instruments enhancement, respectively. *S*_*V*_(*t, m*), *E*_*V*_(*t, m*), and *E*_*I*_(*t, m*) represent the predicted LGF of the target speech, vocals and background interfere or background instruments, respectively.

The back-end pipeline (Figure 3) integrates a TasNet-based DNN model into the ACE sound coding strategy. While the DNN models used in both the front-end and back-end pipelines share a similar architecture, they differ in three aspects: the activation function employed in the encoder, the loss function used in the TCN block, and the output dimensionality of the decoder. Specifically, the back-end model outputs LGFs similar to those generated in the ACE processing chain for each audio segment (i.e., each temporal convolutional window), whereas the front-end model directly estimates time-domain audio signals. Similar to front-end models, two back-end DNN models were trained for distinct source separation tasks: female-male speech separation and vocals—background instruments separation. Both models were optimized using a mean squared error (MSE) loss function, minimizing the discrepancy between the predicted LGF outputs and the corresponding targets generated by the ACE strategy. The initial DNN model was based on Conv-TasNet used in Gajecki et al. (2023). However, we investigated the optimization of Conv-TasNet for the source separation task. To enable the model to generate high-resolution masking functions necessary for source separation, the envelope detector was moved to the decoder, and a new convolutional block was introduced within the decoder. Moreover, this stepwise dimensionality reduction allows for enhanced decoding into the electrodogram domain with reduced interference. Although the electrodogram masker improved DeepACE in speech denoising tasks, it was discarded from the back-end pipeline, as it contributed to higher loss values in source separation tasks. All models were trained using the Adam optimizer (Kingma and Ba, 2014) with an initial learning rate of 0.001, for a maximum of 100 epochs. The architectural specifications of the DNN models that achieved the best performance—both in terms of the loss function and linear correlation coefficients (LCCs) for both pipelines—are provided in Table 2.

### 2.6 Objective instrumental measurements

The Nucleus MATLAB Toolbox, based on MATLAB software (Mathworks, Natick, MA, U.S.A.), was utilized to create the electrodograms of the ACE sound coding strategy for simulation purposes.

#### 2.6.1 Linear correlation coefficient

Objective evaluation was performed using the linear correlation coefficient (LCC), calculated based on the electrodograms produced by each processing pipeline. This analysis enabled the characterization of potential distortions and artifacts introduced by the evaluated pipelines in comparison to the standard ACE electrodograms. LCCs were computed individually for each of the 22 CI channels, followed by averaging across all channels to obtain a global similarity measure.

#### 2.6.2 Front-end and end-to-end enhancement

Due to the non-linear compression applied in the LGF block and other non-linearity introduced by the ACE, the corresponding end-to-end enhancement relative to the front-end enhancement cannot be computed analytically. However, empirical methods allow for the calculation of the equivalent end-to-end enhancement. As expressed in Equation 1, *v*_*F*_[*n*] and *i*_*F*_[*n*] represent the outputs of the front-end DNN model, corresponding to the separated vocals and separated instrumental audio tracks, respectively, with *n* denoting the sample index. These outputs contribute to the calculation of VIR_Enh − F_, which quantifies the enhancement of the vocals relative to the background instruments at the front-end level. The outputs of the end-to-end model are also described in Equation 3, where *E*_*V*_ and *E*_*I*_ denote the electrodograms for the vocals and instruments, respectively. Here, *t* denotes the time frame and *m* the electrode index. These outputs contribute to the calculation of VIR_Enh − E_, which quantifies the enhancement of the vocals relative to the background instruments at the end-to-end level. Similarly, in Equation 2, *v*_*F*_[*n*] and *i*_*F*_[*n*] denote the separated signal and interferer in the front-end model, while Equation 4 refers to the electrodograms of the signal and interferer obtained from the end-to-end model. These signals contribute to the calculation of front-end SIR_Enh-F_ and end-to-end SIR_Enh-E_, respectively.


$$\begin{array}{l} {\text{VIR}}_{\text{Enh-F}}\;\left[\text{dB}\right]=10\cdot \log_{10}\left(\frac{\overset{N}{\underset{n=1}{\sum }}v_{F}{\left[n\right]}^{2}}{\overset{N}{\underset{n=1}{\sum }}i_{F}{\left[n\right]}^{2}}\right), \end{array}$$ (1)

$$\begin{array}{l} {\text{SIR}}_{\text{Enh-F}}\;\left[\text{dB}\right]=10\cdot \log_{10}\left(\frac{\overset{N}{\underset{n=1}{\sum }}s_{F}{\left[n\right]}^{2}}{\overset{N}{\underset{n=1}{\sum }}i_{F}{\left[n\right]}^{2}}\right), \end{array}$$ (2)

$$\begin{array}{l} {\text{VIR}}_{\text{Enh-E}}\;\left[\text{dB}\right]=10\cdot \log_{10}\left(\frac{\overset{T}{\underset{t=1}{\sum }}\overset{M}{\underset{m=1}{\sum }}E_{V_{B}}{\left(t,m\right)}^{2}}{\overset{T}{\underset{t=1}{\sum }}\overset{M}{\underset{m=1}{\sum }}E_{I_{B}}{\left(t,m\right)}^{2}}\right), \end{array}$$ (3)

$$\begin{array}{l} {\text{SIR}}_{\text{Enh-E}}\;\left[\text{dB}\right]=10\cdot \log_{10}\left(\frac{\overset{T}{\underset{t=1}{\sum }}\overset{M}{\underset{m=1}{\sum }}E_{S_{B}}{\left(t,m\right)}^{2}}{\overset{T}{\underset{t=1}{\sum }}\overset{M}{\underset{m=1}{\sum }}E_{I_{B}}{\left(t,m\right)}^{2}}\right). \end{array}$$ (4)

To facilitate a comparison between the front-end and end-to-end pipeline, it was necessary to apply the same amount of VIR_Enh_ to the mixture in both methods. However, the outputs of the front-end DNN and the end-to-end DNN inherently are in different domains. To address this, an VIR_eqi_ was defined, enabling the application of a computationally equivalent VIR_Enh_ to the mixture within the end-to-end pipeline. The VIR_eqi_ used in this study is based on the VIR_Enh_ used in Tahmasebi et al. (2023) and is defined as


$$\begin{array}{l} {\text{VIR}}_{\text{eqi}}\;\left[\text{dB}\right]=10\cdot \log_{10}\left(\frac{\overset{T}{\underset{t=1}{\sum }}\overset{M}{\underset{m=1}{\sum }}E_{V_{F}}{\left(t,m\right)}^{2}}{\overset{T}{\underset{t=1}{\sum }}\overset{M}{\underset{m=1}{\sum }}E_{I_{F}}{\left(t,m\right)}^{2}}\right), \end{array}$$ (5)

where *E*_*V*_*F*__ and *E*_*I*_*F*__ denote the outputs of the front-end model, while *v*_*F*_ and *i*_*F*_ are processed using the ACE sound coding strategy. In this context, *t* and *m* correspond to the time frame and frequency band, respectively. Table 3 shows the front-end and end-to-end equivalent VIR_Enh_. Note that due to non-linearity of the sound coding strategy, increments 1 dB steps increase in front-end does not lead to the same increase in end-to-end. It is worth mentioning that VIR_Enh − F_ = 0 is equal o VIR_Enh − E_ of 0.17 dB which shows that the sound coding strategy emphasizes the vocals. Similar to vocals enhancement using VIR_Enh_, we performed target speech enhancement by applying SIR_Enh_ both in front-end and end-to-end pipeline. SIR_Enh_ can be configured either to completely suppress the competing talker or, depending on the task, such as spatial awareness, to an optimal value that balances speech intelligibility and the preservation of spatial cues.

**Table 3:** The equivalent enhancement for front-end and end-to-end pipeline.

**VIR enhancement method**	**Equivalent VIRs**								
Front-end	0 dB	1 dB	2 dB	3 dB	4 dB	5 dB	6 dB	7 dB	8 dB
End-to-end	0.17 dB	0.28 dB	0.41 dB	0.58 dB	0.74 dB	0.96 dB	1.28 dB	1.53 dB	1.95 dB

### 2.7 Perceptual experiments

Two perceptual experiments were conducted to evaluate the effect of end-to-end and Front-end CI sound coding pipeline and enhancement models in speech understanding and music perception and appreciation in CI users. Prior to the beginning of the perceptual experiments, individual fitting parameters were retrieved from the Cochlear database and imported into the clinical speech processor used for the experiments.

### 2.8 Speech understanding test

Speech intelligibility was assessed using the HSM sentence test, mixed at different SIRs for male and female voice as target. In the first condition, speech intelligibility using a female target with a male voice as the competing talker was measured. In the second condition, the target and the competing talker were switched allowing to measure the speech intelligibility with a male speaker as target and a female voice as the competing talker. Prior to the onset of testing, each participant underwent three training sessions to determine their individual optimal input SIR using ACE algorithm. All conditions of the speech intelligibility test were subsequently conducted at this individualized input SIR for each subject. Both conditions were performed at the same SIR for each subject. Finally, to maximize speech understanding, the parameter SIR_Enh_ was set to a high value, aiming to completely suppress the interferer.

### 2.9 Music perception and appreciation questionnaire

10 Music excerpts from Buyens dataset (Buyens et al., 2014) and the iKala dataset (Chan et al., 2015) were used in the music perception and appreciation questionnaires. In the first questionnaire, the subjects were asked to rate the clarity of the singing voice “how clear is the singing voice.” In the second questionnaire, participants rated their overall music enjoyment as “how is your overall music enjoyment.” Each music excerpt, 10 s in duration, was processed 3 times using the 3 electrodogram generating method employed in this study: the classic ACE, the front-end pipeline, and the end-to-end pipeline. The front-end and end-to-end approach apply 6 dB, and 1.28 dB VIR to the mixture, respectively. Given that applying an enhancement to a track results in an overall increase in mixture intensity and perceived loudness, we implemented a 6 dB VIR_Enh − F_ by applying a 3 dB gain to the vocals and a 3 dB attenuation to the background instruments. Similar procedure were utilized to apply VIR_Enh − E_. Table 4 shows the details of the music excerpts used in the questionnaires. Participants were instructed to rate each music excerpt on a scale from 1 to 15, with 15 representing the highest perceived pitch.

**Table 4:** Singing music excerpts employed in the music perception and appreciation questionnaires.

**ID**	**Song name**	**Vocals**	**Piano**	**Guitar**	**Bass**	**Drums**
M1	Before I Go (Papermouth)	+	+	+	–	–
M2	21045 chorus	+	–	–	–	
M3	Hallelujah (Leonard Cohen)	+	+	–	–	–
M4	00042 IKALA	+	–	–	–	
M5	Hey Jude (The Beatles)	+	–	+	+	+
M6	0005 3 mic2	+		–	–	
M7	Michel (Anouk)	+	–	+	+	–
M8	45387 verse	+	–	–	–	
M9	Dock of the Bay (Otis Redding)	+	+	+	+	+
M10	31126 chorus	+	–	–	–	+

### 2.10 Statistical analysis

Inferential data analysis was performed using the R statistical software (via RStudio), employing the packages lme4, lmerTest, and emmeans. Perceptual evaluation results were analyzed using linear mixed-effects models, with the HSM test scores and questionnaire responses as outcome variables, and CI participants specified as a random factor. The Shapiro–Wilk test was applied to assess the normality of the distributions gathered from the perceptual evaluation data. In order to evaluate whether significant differences exist in the mean outcomes across the three electrodogram generating methods (ACE, End-to-end pipeline, and Front-end pipeline), a repeated measures analysis of variance (ANOVA) was conducted for normally distributed data, and a non-parametric Friedman rank sum test was applied to non-normally distributed data. *Post hoc* tests were performed using the emmeans package (version 1.10.2) for normally distributed data, and multiple pairwise comparisons using Wilcoxon signed-rank tests for non-normally distributed data. Statistical significance was determined using a threshold of *p* < 0.05, with p-values adjusted for multiple comparisons using the Bonferroni correction. The null hypothesis was rejected for all comparisons meeting this criterion.

## 3 Results

### 3.1 Objective instrumental measurements

**Figure 4:**
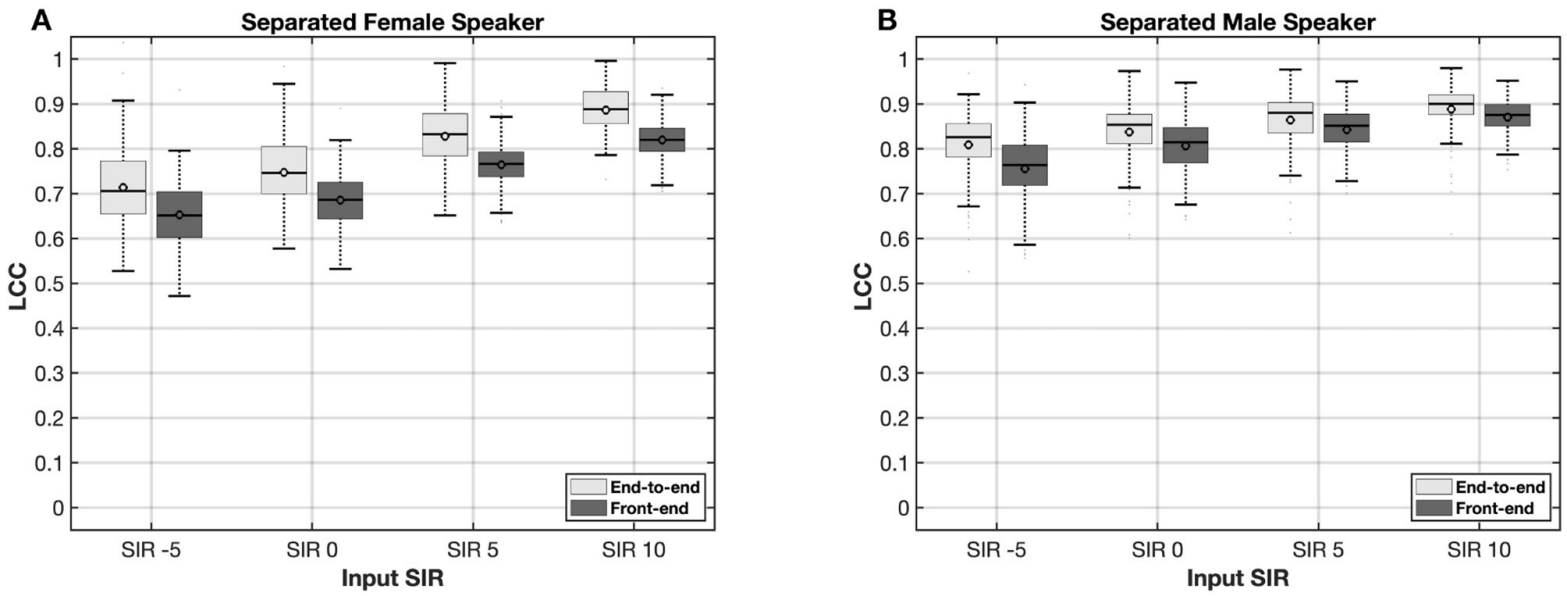
Linear correlation coefficients (LCCs) at various input signal-to-interference ratios (SIRs) for separated female **(A)** and male **(B)** speakers with end-to-end and front-end pipelines.

**Figure 5:**
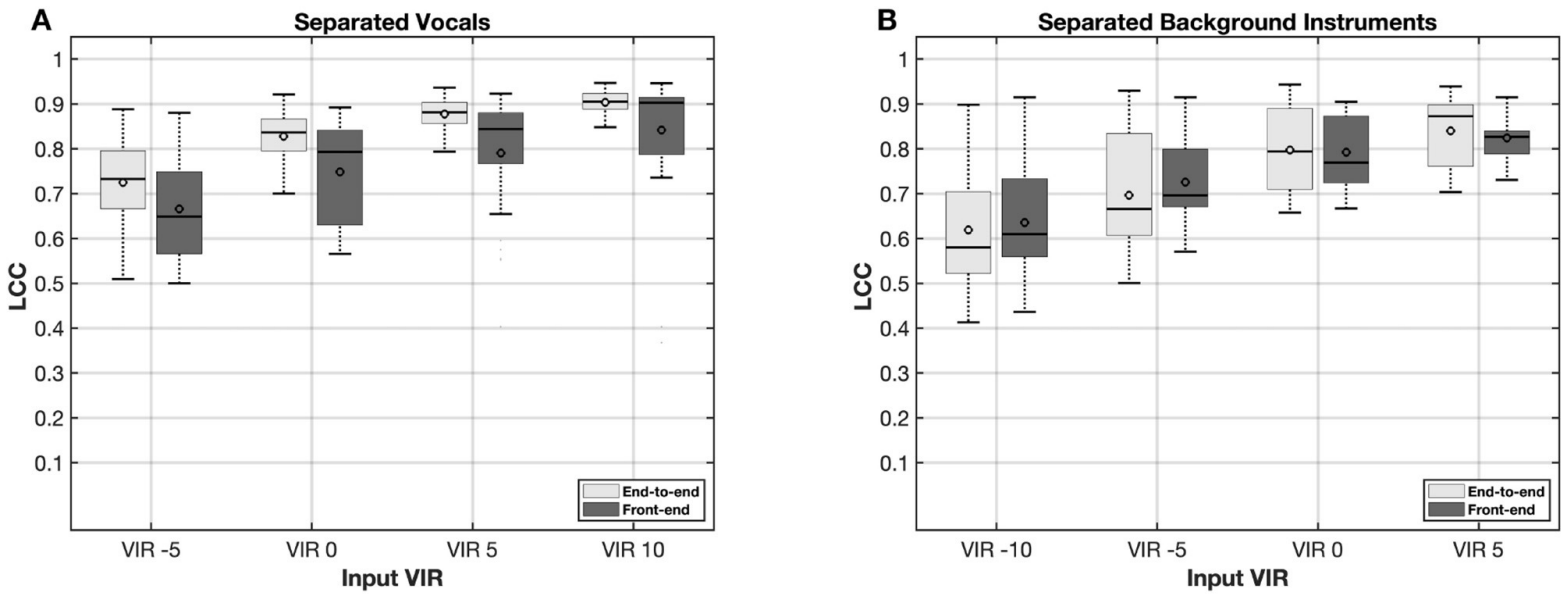
Linear correlation coefficients (LCCs) at various input vocals to instruments ratios (VIRs) for separated vocals **(A)** and background instruments **(B)** using end-to-end and front-end pipelines.

Figures 4, 5 present the results of the LCC analysis of the end-to-end and front-end pipeline models. The coefficients are presented as a mean value across all electrodes for each single audio. The results of the speech separation model for male and female voice as target for 4 different input SIR are shown in Figures 4A, B, respectively. Across all input SIRs, the end-to-end pipeline provides a higher LCCs compared to the Front-end pipeline. The results obtained from the female voice are slightly lower than the male voice for both end-to-end and front-end pipeline. This might be due to the quality difference in the testing dataset in which the female speaker showed a lower clarity. This explains the greater improvements by increasing input SIRs for the female voice compared to the male voice.

The results of the singing music source separation pipeline for both vocals and background instruments across four different input VIRs are presented in Figures 5A, B, respectively. Consistent with the results observed in the male–female voice separation pipeline, the end-to-end pipeline for singing music source separation yielded higher LCCs compared to the front-end pipeline. While the end-to-end pipeline exhibited lower variability than the front-end approach, both pipelines demonstrated greater variability overall compared to the male–female source separation tasks. Among all conditions, the LCCs obtained for the background instrument separation were the lowest and exhibited the highest degree of variability.

### 3.2 Speech understanding

**Figure 6:**
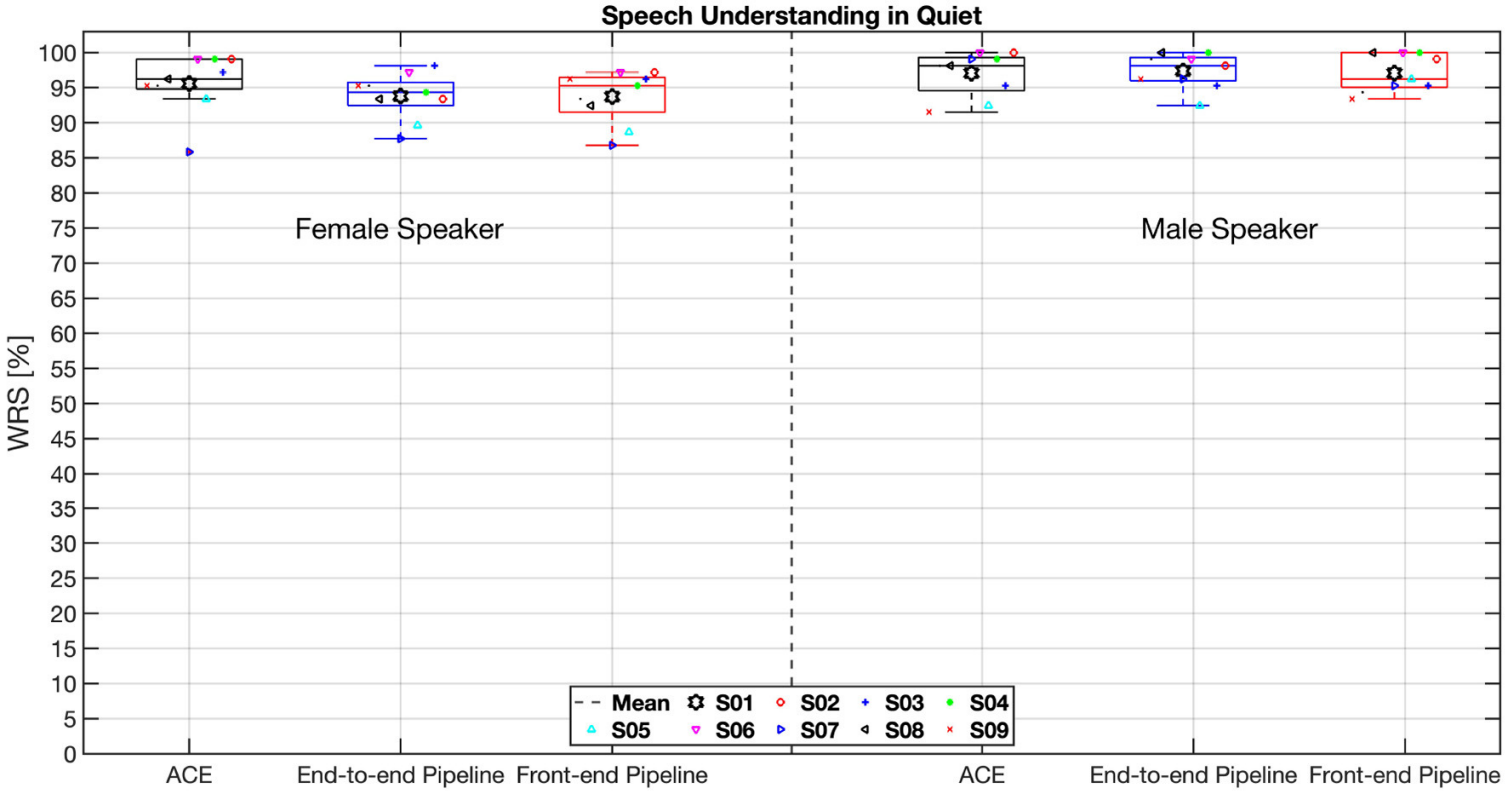
Individual and average word recognition scores (WRS) from the HSM test in quiet conditions are presented for nine participants, with a male speaker as the target in the left three box plots and a female speaker in the right three box plots. Each box plot illustrates the interquartile range (25th–75th percentiles), with the median indicated by a horizontal line and the mean represented by a filled black hexagram. Outliers, defined as values exceeding 1.5 times the interquartile range above the upper quartile or below the lower quartile, are marked with red plus signs. Colored symbols indicate individual participant scores. WRS, word recognition score, expressed as a percentage.

Figure 6 shows the percentage of correctly understood words in quiet for the three electrodogram-generating methods, using both a female speaker (left three box plots) and a male speaker (right three box plots) as target voices. Non-parametric Friedman tests conducted separately for the female and male speaker conditions revealed no statistically significant differences among the tested methods (*p* = 0.1211 and *p* = 0.8984, respectively). The mean scores for the female speaker were consistently lower than those for the male speaker across all three methods, which may be attributed to the relatively lower quality and clarity of the female speech material used in the word recognition tests.

**Figure 7:**
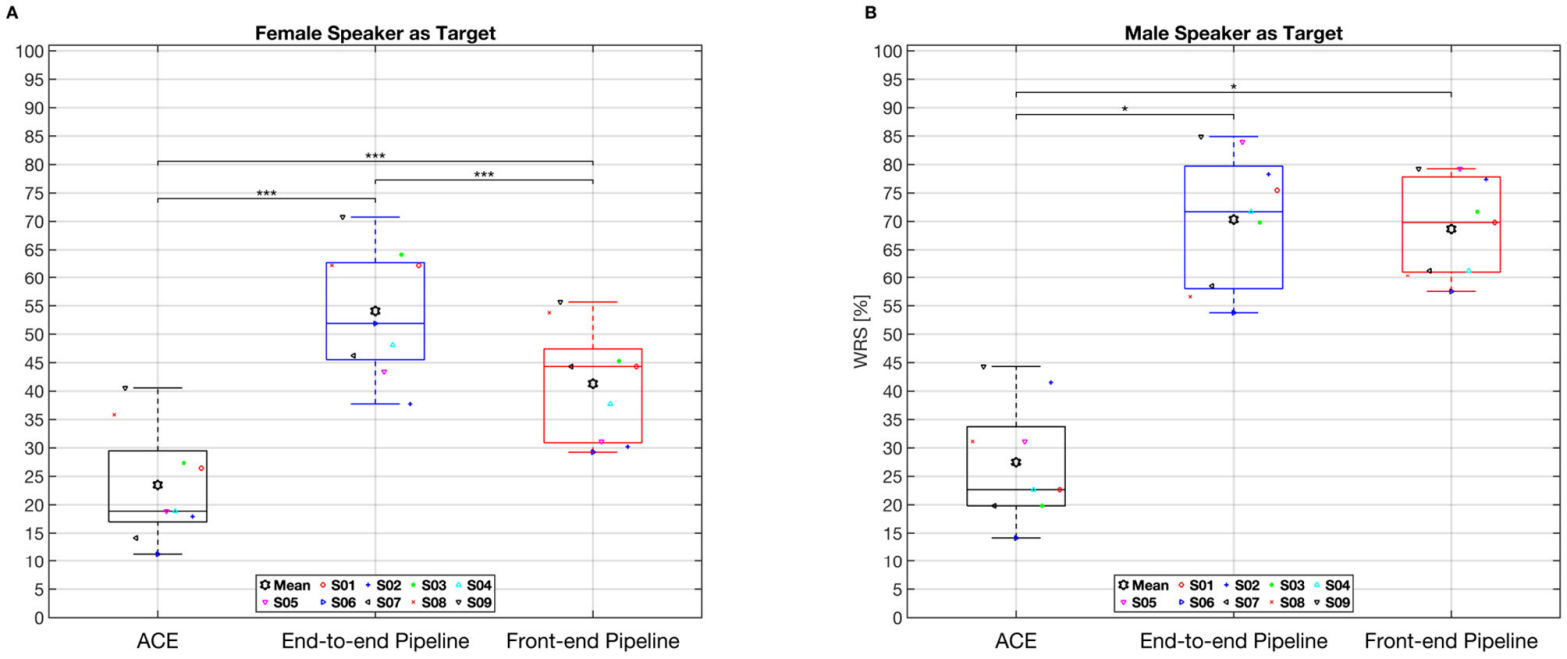
Individual and average word recognition scores (WRS) obtained from the HSM test are shown for nine participants, with a female speaker as the target in the left panel **(A)** and a male speaker in the right panel **(B)**. Box plots display the interquartile range (25th–75th percentiles), with the median represented by a horizontal line and the mean indicated by a filled black hexagram. Outliers, defined as values exceeding 1.5 times the interquartile range above the third quartile or below the first quartile, are marked by red plus signs. Colored symbols denote individual participant scores. WRS, word recognition score, expressed as a percentage. Levels of statistical significance are denoted by asterisks: *indicates *p* < 0.05, **indicates *p* < 0.001.

Participants' performance on the speech understanding test in the presence of a competing voice is presented in Figure7. Figure 7A displays the results of speech understanding when the target speaker was female and the interfering speaker was male. Conversely, Figure 7B illustrates the percentage of correctly recognized words when the target speaker was male and the interfering voice was female. In both conditions, the end-to-end pipeline provided the highest WRS, with mean values of 59% and 71% for the female and male target voices, respectively. As results obtained from female voice target condition were normally distributed, a repeated measures ANOVA was performed and revealed a significant difference among the three tested electrodogram generating methods in the WRS results, *F*(2, 18) = 113.42, *p* < .0001. A followed up with *post-hoc* Bonferroni adjusted pairwise comparisons revealed significant difference among all comparison with End-to-end pipeline outperforming both ACE and the Front-end pipeline, *p* < .001 for all three comparisons. In contrast, as the data from the male voice target condition did not follow a normal distribution, a non-parametric Friedman test was conducted, followed by Bonferroni-adjusted *post hoc* tests. The Friedman test revealed a statistically significant effect across the three conditions, χ^2^(2) = 13.56, *p* = 0.0011. *Post hoc* analyses indicated significant differences between the ACE strategy and both the End-to-end pipeline (*p* = 0.022) and the Front-end pipeline (*p* = 0.023). No significant difference was observed between the End-to-end and Front-end pipeline results.

### 3.3 Music perception and appreciation

**Figure 8:**
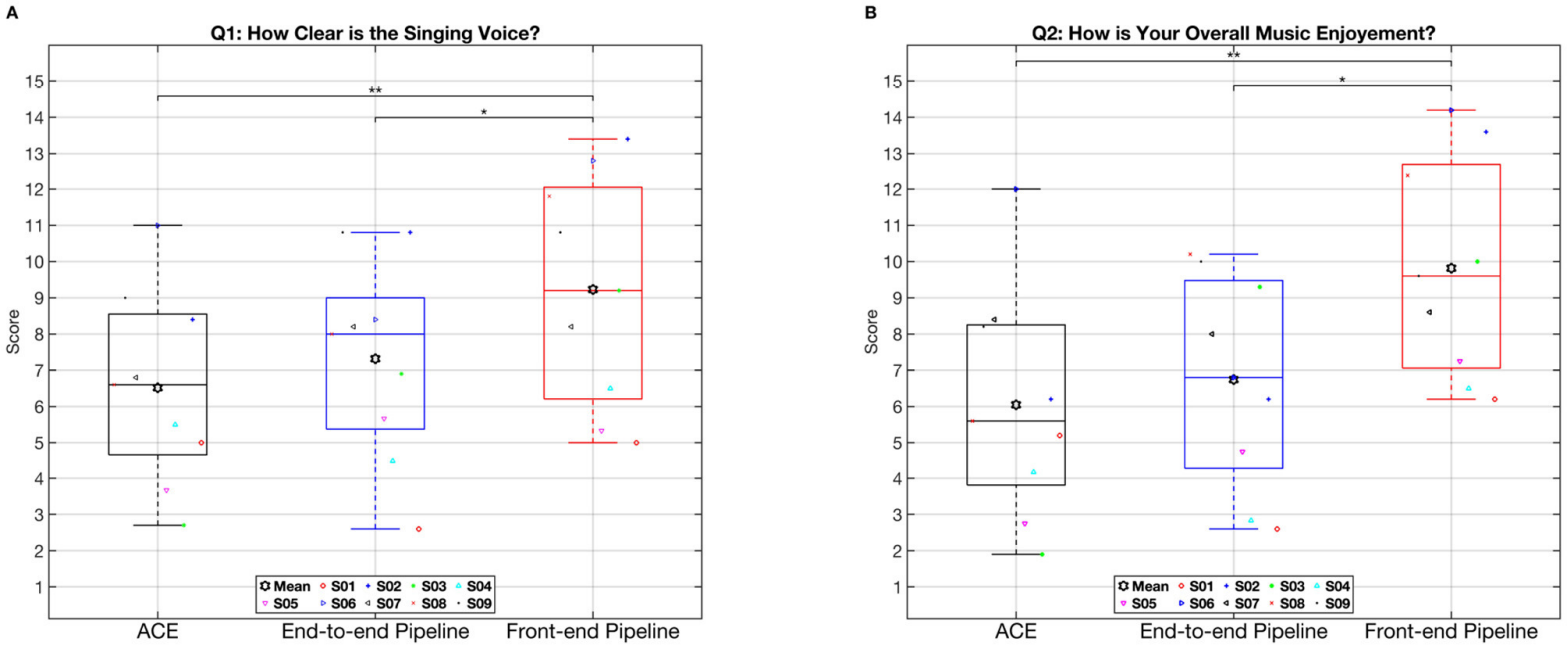
Participant responses from the musicperception and appreciation questionnaire. The left panel **(A)** shows responses to the question, “How clear is the singing voice?”, while the right panel **(B)** displays responses to “How is your overall music enjoyment?”. The red line and the filled black hexagram represent the median and mean values, respectively. Individual participant responses are depicted using colored symbols. Levels of statistical significance are denoted by asterisks: *indicates *p* < 0.05, **indicates *p* < 0.01.

Figures 8A, B present the subjective preferences of nine CI users based on responses to the music perception and appreciation questionnaire, evaluating the three tested electrodogram generation methods. Subjective perception was assessed using two questionnaires: “clarity of the singing voice” (left panel, Figure 8A) and “overall music enjoyment” (right panel, Figure 8B). In both questionnaires, the front-end pipeline consistently yielded higher mean ratings compared to ACE and the End-to-end pipeline. A repeated measures ANOVA for the first questionnaire, “How clear is the singing voice?”, revealed a statistically significant difference among the three excitation pattern generating methods, *F*(2, 18) = 7.89, *p* = 0.0041. Subsequent *post hoc* comparisons indicated that the front-end pipeline significantly outperformed both ACE (*p* = 0.004) and the End-to-end pipeline (*p* = 0.045) in terms of perceived clarity. No additional statistically significant differences were observed across the remaining comparisons. Since the data gathered from the second questionnaire were also normally distributed, we a performed second ANOVA which revealed a significant difference between tested electrodogram generation methods (*F*(2, 18) = 6.96, *p* = 0.006). A *post-hoc* Bonferroni adjusted pairwise comparison revealed that Front-end algorithm improved significantly the overall enjoyment of the music excerpts with respect to the clinical ACE and the End-to-end algorithm (*p* = 0.008 and *p* = 0.034, respectively). No significant difference between the mean values obtained from the ACE and End-to-end algorithm was observed.

## 4 Discussion

In this study, front-end and end-to-end source separation pipelines for female-male speech and singing music were investigated for CI users, and their performances were compared. Similar to speech understanding in noise, music perception and appreciation with CIs has been a major focus of research in the CI field. To address these challenges, DNN-based approaches have gained attention and have shown promising results in front-end and end-to-end for both music and speech. Zheng et al. (2021) introduced a DNN-based model (NNACE) to simulate the envelope detection of the classic ACE sound coding strategy aiming at enhanced noise robustness and better representation of fine temporal structures. Perceptual evaluations conducted with vocoded speech in NH listeners demonstrated significant in speech reception thresholds compared with the classic ACE strategy. Huang et al. (2023) focused on extending the envelope detection with N-of-M band selection using a DNN-based approach (ElectrodeNet). Gajecki et al. (2023) introduced DeepACE, an end-to-end deep leaning based coding strategy that simultaneously functions as denoising algorithm. Perceptual evaluation in CI users demonstrated significant improvements in noisy speech understanding with DeepACE over classic ACE and front-end pre-processing algorithms.

Since classic sound coding strategies use rule based signal processing algorithms, they have the advantage of performing very well in quiet conditions. However, in noisy conditions such as cafeteria noise or a speech masker the performance of classic sound coding strategies drops drastically. Speech understanding in the presence of a speech masker is particularly challenging for CI users, as it demands better enhanced abilities such as better pitch perception and auditory stream segregation, along with the engagement of higher-level cognitive processes like selective attention (Başkent and Gaudrain, 2016). In contrast, DNN models are data-driven approaches that learn from the training datasets on which they are trained. These models incorporate a stochastic element, and although they can produce highly accurate outputs, the internal reasoning behind their decisions is often opaque. This lack of transparency may lead to the introduction of artifacts, which could negatively impact sound perception for CI users, particularly in quiet listening conditions, when compared to traditional rule-based sound coding strategies. However, perceptual evaluation results from speech understanding tasks in quiet environments revealed no significant differences between the conventional ACE strategy and the two DNN-based approaches used in our study. Both the end-to-end and front-end pipelines yielded comparable speech understanding scores (%WRS) for female and male target speaker.

In the speech understanding test, both the end-to-end and front-end approach outperformed the classic ACE for both female and male speaker as target. The front-end pipeline achieved a significant improvement in word recognition scores (WRS) of approximately 20% and 40% for female and male targets, respectively. These results are consistent with the findings in hearing aids reported by Diehl et al. (2023), where the use of a front-end DNN model led to a significant improvement of approximately 4 dB in speech reception threshold across three different background noises, including restaurant and traffic noise. However, the end-to-end approach presented in the current work provided significantly better results for the female speaker compared to the front-end approach. The results form perceptual evaluation are consistent with the objective instrumental measurements where the end-to-end pipeline provided consistently better LCCs compared to the front-end pipeline for both female and male speaker across various input SIRs. The results from the front-end approach are consistent with Goehring et al. (2019) where DNN-based FFT masking model was employed as a front-end to enhance the acoustic signal. The results from that study demonstrated significant improvements in both objective instrumental measurements and perceptual evaluations in CI users. The results from end-to-end compare to front-end pipeline are consistent with Gajecki et al. (2023) where the end-to-end approach outperformed the front-end approach in speech denoising tasks using various noises such as speech shape stationary noise and non-stationary babel noise.

Contrary to the speech understanding test, the results from the music questionnaires were not consistent with the objective instrumental measurements. The questionnaire ratings obtained from the front-end approach were significantly better than those from the end-to-end and classic ACE approaches, for both the clarity of the singing voice (Q1) and overall music enjoyment (Q2). The results from the front-end algorithm are consistent with previous work in the STFT domain (Tahmasebi et al., 2020; Gajęcki and Nogueira, 2018). Similar improvements in music appreciation were reported by Gauer et al. (2022), who proposed a front-end DNN-based model enabling individualized remixing of audio content through the isolation and adjustment of specific sound sources, such as bass or midrange frequencies. Likewise, Tahmasebi et al. (2020) employed a front-end DNN-based system to separate vocals from background instruments, thereby allowing CI users to apply a VIR and remix music tracks according to personal preference.

However, to the best of our knowledge, this study is the first to investigate an end-to-end or back-end music source separation method for CI users. To address these results, we explored all steps in both the end-to-end and front-end pipelines. In contrast to the front-end pipeline, where the separated background instrument tracks are first remixed with the vocals before entering the sound coding strategy, the end-to-end pipeline passes the vocals and background instruments separately through the sound coding strategy. However, the sound coding strategy and many of its signal processing blocks have been developed and optimized primarily for voice (Tahmasebi et al., 2023), rather than for background instruments. As a result, while it is well capable of generating optimal electrodograms for vocal tracks, it fails to do so for background instruments. One of the first steps in creating a data-driven model such as a DNN is proper data preparation. DNN models learn from examples and aim to generalize what they have learned to new inputs. If they are initially trained on suboptimal examples, the resulting performance will also be suboptimal. In the context of an end-to-end or back-end music source separation, this means that the DNN model was trained on a non-optimal dataset, which led to non-optimal results in the perceptual evaluation. Although, based on objective instrumental measurements, the end-to-end model was capable of predicting electrodograms similar to those generated by ACE and even achieve results comparable to the front-end approach, it was ultimately outperformed by the front-end strategy in the music questionnaires. We argue that for a successful and well-perceived end-to-end music source separation pipeline for CI users, a key missing element is a sound coding strategy specifically developed and optimized for background instruments. Such a strategy would be capable of providing the end-to-end DNN model with an optimal training dataset.

Compared to end-to-end excitation pattern enhancement approaches Gajecki et al. (2023), front-end source separation pipeline enables control over individual sound sources within an audio mixture, allowing for customized remixes of the constituent components for CI users. For example, in noisy environments, such separation can be used to retain partial background noise to preserve spatial awareness. In music applications, it allows for the enhancement of specific elements such as vocals (Tahmasebi et al., 2020) or targeted frequency ranges like bass and midrange (Althoff et al., 2024), even in real time for individual CI users. However, compared to end-to-end excitation pattern enhancement approaches, such as those proposed in Gajecki et al. (2023), which aim to predict a single clean, denoised, and de-reverberated target signal, front-end source separation demands higher resolution and a larger DNN model to effectively estimate all individual sound sources. All front-end models used in the present study are larger than the end-to-end models employed by Gajecki et al. (2023), even though both are based on the same architecture, namely Conv-TasNet (Luo and Mesgarani, 2018). This, in turn, increases the demand for computational resources and presents a challenge for real-time implementation in CI sound processors.

CI deep learning–based source separation approaches, both front-end and end-to-end processing that integrate sound coding strategies face several potential limitations, including system complexity, high computational demands (often associated with increased power consumption and reduced battery life), and algorithmic latency. However, in all three of these aspects, the end-to-end pipeline offers advantages over front-end solutions. First, it reduces redundancy in the processing pipeline. Second, it integrates the sound coding strategy, at least partially, directly into the DNN model. This integration allows the end-to-end approach to lower computational load and power consumption by minimizing unnecessary front-end processing. In addition, it provides better control over target signal enhancement, such as enhancing vocals within a mixture, since the enhancement occurs at the final stages of the pipeline and bypasses many nonlinear signal processing blocks earlier in the chain.

## 5 Conclusion

This study investigated the integration of DNNs with CI sound coding strategies for speech and music separation tasks. Specifically, the study compared a CI sound coding front-end with an end-to-end pipeline to generate electric excitation patterns. These excitation patterns were compared to those produced by a conventional CI sound coding strategy. All processing approaches were evaluated using objective instrumental measurements and subsequently assessed in perceptual evaluations involving nine CI users. In speech separation tasks, end-to-end pipeline outperformed the front-end pipeline in both objective instrumental measurements and speech understanding tests in CI users. In contrast, for music source separation, although the end-to-end pipeline demonstrated a better performance based on objective instrumental measurements, the front-end pipeline received higher subjective ratings in the questionnaire responses regarding both the clarity of the singing voice and overall music enjoyment. The findings of this study demonstrate the potential of the DNN-based end-to-end pipeline for effective speech and music source separation. However, for end-to-end music source separation applications, it is essential to first develop a CI sound coding strategy specifically optimized for the accurate representation of background instruments. Ideally, separate CI sound coding strategies tailored to the singing voice and background instruments should be used to generate appropriate training datasets for the DNN-based end-to-end model.

## Data Availability

The original contributions presented in the study are included in the article/supplementary material, further inquiries can be directed to the corresponding author.
